# RNA 5-methylcytosine regulates YBX2-dependent liquid-liquid phase separation

**DOI:** 10.1016/j.fmre.2021.10.008

**Published:** 2021-12-08

**Authors:** Xiuzhi Wang, Mengke Wang, Xinyuan Dai, Xiao Han, Yu Zhou, Weiyi Lai, Liyuan Zhang, Ying Yang, Yusheng Chen, Hailin Wang, Yong-Liang Zhao, Bin Shen, Yuhan Zhang, Ying Huang, Yun-Gui Yang

**Affiliations:** a Key Laboratory of Genomic and Precision Medicine, Collaborative Innovation Center of Genetics and Development, College of Future Technology, Beijing Institute of Genomics, Chinese Academy of Sciences, Beijing 100101, China; b Department of General Surgery, Shanghai Key Laboratory of Biliary Tract Disease Research, State Key Laboratory of Oncogenes and Related Genes, Xinhua Hospital, Shanghai Jiao Tong University, Shanghai 200092, China; c State Key Laboratory of Reproductive Medicine, Nanjing Medical University, Nanjing 211166, China; d China National Center for Bioinformation, Beijing 100101, China; e University of Chinese Academy of Sciences, Beijing 100049, China; f Sino-Danish College, University of Chinese Academy of Sciences, Beijing 100049, China; g Institute of Stem Cell and Regeneration, Chinese Academy of Sciences, Beijing 100101, China; h Research Center for Eco-Environmental Sciences, Chinese Academy of Sciences, Beijing 100085, China

**Keywords:** RNA modification, RNA m^5^C, YBX2, Phase separation, Liquid droplet

## Abstract

5-Methylcytosine (m^5^C) is one of the most prevalent internal modifications of messenger RNA (mRNA) in higher eukaryotes. Here we report that Y box protein 2 (YBX2) serves as a novel mammalian m^5^C binding protein to undergo liquid-liquid phase separation (LLPS) both *in vivo* and *in vitro,* and this YBX2-dependent LLPS is enhanced by m^5^C marked RNA. Furthermore, the crystal structure assay revealed that W100, as a distinct m^5^C binding site of YBX2, is critical in mediating YBX2 phase separation. Our study resolved the relationship between RNA m^5^C and phase separation, providing a clue for a new regulatory layer of epigenetics.

## Graphical abstract

**Figure:**
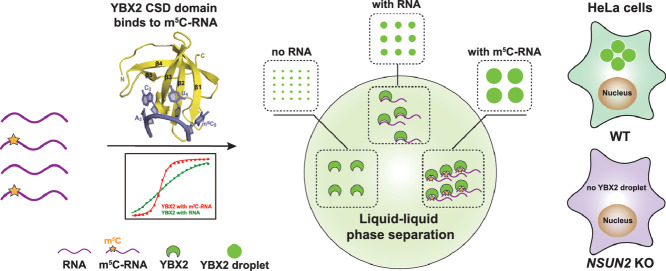

## 1 Introduction

The dynamic and reversible RNA methylation pioneers a new frontier area of epitranscriptomics. As one of the most prevalent and abundant RNA modifications, RNA 5-methylcytosine (m^5^C) has been revealed to play roles in various RNA processing and biological processes, such as gametogenesis and embryogenesis [1], [2], [3], [4], tumorigenesis and migration [5], [6], [7]. With the rapid development and broad application of sequencing technologies, RNA bisulfite sequencing (RNA-BisSeq) established in 2012 has achieved single base-resolution for m^5^C identification [8]. RNA m^5^C is a dynamic and reversible modification regulated by its related enzymes, including m^5^C methyltransferases, demethylase, and binding proteins [9]. RNA m^5^C writers include NOL1/NOP2/sun domain (NSUN) family members, DNA methyltransferase (DNMT) family members, and tRNA specific methyltransferase (TRDMT) family members [10]. TET2 of the TET family and ALKBH5 of the ALKBH family, the two enzymes first identified as DNA 5mC demethylase, have been confirmed to act as RNA m^5^C erasers to remove m^5^C modifications from RNA. ALYREF (Aly/REF Export Factor) and Y box protein 1 (YBX1) are two mRNA m^5^C readers known so far [11].

Studies about ALYREF and YBX1 unmasked their regulatory roles in the export and stability of m^5^C marked mRNA, respectively [3,12,13]. Interestingly, as the members of the Y-box protein family, YBX1, YBX2, and YBX3 are all structurely characterized by the alanine/proline (A/P)-enrich N terminal domain, the variable C-terminal domain (CTD), and the highly conserved cold shock domain (CSD) [14]. According to the previous study, human YBX1 prefers to recognize m^5^C-modified mRNAs through π-π interactions with a critical tryptophan residue in its CSD [3]. The significant sequence similarity in CSD implies a similar function of YBX2 and YBX3 to YBX1, which however remains unclear till present.

It is worth noting that liquid-liquid phase separation (LLPS) has emerged as a new layer of epigenomics. LLPS organizes many non-membrane-bound compartments in cells, which could drive germ granules formation, especially ribonucleoprotein (RNP) granules formed through weak promiscuous interactions between RNA-binding proteins (RBPs) and/or RNAs [15]. LLPS is indicated to participate in a variety of biological processes, such as cell division [16,17], RNA storage [18,19], and stress response [20,21]. RNA m^6^A binding proteins YTHDF1, YTHDF2, and YTHDF3 were reported to form LLPS both *in vivo* and *in vitro,* which could be enhanced by multiple-m^6^A-modified mRNAs [22]. METTL3-mediated m^6^A methylation could promote stress granule (SG) formation via increasing *miR-335* maturation and shows the potential therapeutic strategy for Acute Ischemic Stroke (AIS) [23]. However, it is still elusive whether m^5^C regulates the formation of phase separation. YBX2 has the potential to recognize m^5^C-modified RNA as a homologous protein of YBX1. Specifically, YBX2 contains a low complexity domain (LCD), which is necessary for the formation of phase separation [24], suggesting a LLPS formation potential for YBX2.

To address whether YBX2 could regulate the formation of phase separation by recognizing m^5^C-modified mRNA, we solved the crystal structure of YBX2 CSD complexed with m^5^C modified RNA. Importantly, we found a strong correlation between RNA m^5^C modification and LLPS driven by YBX2 both *in vivo* and *in vitro*. Moreover, YBX2-dependent phase separation is regulated by m^5^C modification.

## 2 Materials and methods

### 2.1 Materials

#### 2.1.1 Mice

The wild-type C57BL/6 of 6–8 weeks were purchased from SPF (BeiJing) Biotechnology Co., Ltd. Mice were euthanized by cervical dislocation. Procedures were approved by the Animal Experimental Ethical Inspection Form of Beijing Institute of Genomics/China National Center for Bioinformation.

#### 2.1.2 Cell lines

Cells used in this study, including GC-2spd (ts) cells, HEK293T cells, HeLa cells, and *NSUN2* knock-out (KO) HeLa cells (constructed by our laboratory as previously described [3]), were cultured in standard Dulbecco's Modified Eagle Medium (Gibco) supplemented with 10% fetal bovine serum (Biological Industries), 1% non-essential amino acid (Gibco), and 1% penicillin/streptomycin (Beyotime Biotechnology) at 37 °C in standard humidified 5% CO_2_ cell culture incubators.

### 2.2 Methods

#### 2.2.1 In vivo RNA pulldown assay with MS or western blot analysis

Total testis was homogenized with lysis buffer (100 mM NaCl, 10 mM Tris–HCl pH 7.6, 2 mM EDTA, 0.5% NP-40, 0.5 mM DTT, 1x Proteinase inhibitor, 0.4 U/ul RNase inhibitor). Total protein lysis was precleared by centrifuge at 12, 000 g for 10 min at 4 °C. DNA was removed by DNase I treatment. The sample was pre-cleared by adding 20 μl streptavidin-conjugated magnetic beads, 250 μg yeast tRNA, 0.4 U/μL RNase inhibitor and rotating for 1 h at 4 °C. 1.5 μM biotin labeled RNA probe with or without m^5^C modification (biotin-CGACGGCGGUC(m^5^C) UC (m^5^C) GGGAACC) was added to pre-cleared testis protein and rotating for 2 h at 4 °C. At the same time, 100 μl streptavidin-conjugated magnetic beads were blocked by 0.2 mg/mL BSA, 50 μg/mL yeast tRNA, 0.2 U/mL RNase inhibitor, 1 × Proteinase inhibitor and rotating for 2 h at 4 °C. Add blocked beads to protein extract with 1 μM biotinylated bait RNA and rotate for 30 min at room temperature. The unbound solution was removed and the beads were washed 5 times with wash buffer (100 mM NaCl, 150 mM KCl, 1.5 mM MgCl2, 10 mM Tris–HCl pH7.6, 0.4 mM EDTA, 0.05% NP-40, 0.5 mM DTT, 1x Proteinase inhibitor, 0.4 U/ul RNase inhibitor). The bound proteins were eluted with 30 μl 1 × NuPAGE LDS sample buffer by boiling at 95 °C for 10 min. The sample was separated by SDS-PAGE gel and ready for Mass Spectrum (MS) and Western Blotting detection (WB).

#### 2.2.2 Protein purification in *E. coli* cells

DNA fragments encoding the YBX2 CSD (residues 85–165 and 85–177) were cloned into the pET28-SMT3 vector containing an N-terminal His6-SUMO tag. I92T/Q93K, W100F, and W100F/Y173F mutants were generated using a Site-Directed Mutagenesis Kit according to the manufacturer's instructions. The recombinant proteins were expressed in *Escherichia coli* BL21 (DE3). The cells were grown at 37 °C to an OD600 of 0.6 and induced with 0.2 mM isopropylthiogalactoside overnight at 18 °C. The cells were collected by centrifugation at 4000 r.p.m. for 15 min and lysed using French Press (JNBIO). The proteins were purified using a HisTrap HP column (Cytiva), followed by the cleavage of the tag using Ulp1 protease. The proteins were further purified with a HisTrap S column (Cytiva) and Superdex G75 Hiload 16/60 column (Cytiva). The fractions containing the target proteins were pooled and concentrated to 10 mg/mL in a buffer containing 10 mM Tris–HCl, pH 8.0, 100 mM NaCl, and 1 mM dithiothreitol.

DNA fragments encoding the MusYBX2 full length (residues 1–359), N domain (residues 1–84), CSD (residues 85–196), and C domain (residues 197–359) were cloned to the pET28-SMT3 vector containing an N-terminal His6-SUMO tag and RFP tag, respectively. W101F, Y174F, and W101F/Y174F mutants were generated using a Site-Directed Mutagenesis Kit according to the manufacturer's instructions. The recombinant proteins were expressed and purified as described above.

#### 2.2.3 Electrophoretic mobility shift assay (EMSA)

WT or W101F YBX2 protein was diluted to a series of concentrations of 0 μM, 0.1 μM, 0.2 μM, 0.4 μM with 1x binding buffer (50 mM Tris–HCl, pH 7.5, 100 mM NaCl, 0.4 mM EDTA, 0.1% NP-40, and 40 U/mL RNase inhibitor, 1 mM DTT, 50% glycerol, 5 ng/mL BSA). RNA probes with (biotin-AGAGUGAAACUCCAUCUCAAAC) or without m^5^C (biotin-AGAGUGAAACU (m^5^C) (m^5^C) AUCUCAAAC) modifications were heated at 95 °C for 2 min to disrupt the RNA secondary structure. 1 μL RNA probe (final concentration is 30 nM) and 1 μL purified protein (0 nM, 10 nM, 20 nM, 30 nM, 40 nM final concentration, respectively) were mixed and incubated on ice for 30 min. Then, 1 μL glutaraldehyde (0.2% final concentration) was added into the mixture with a gentle mix and incubated on ice for 15 min. After adding 4 μL HI-DENS TBE SMPL BUF (5 × ) (Thermo Fisher), samples were separated on 6% TBE gel at 80 V for 30 min, followed by transferring the RNA and protein complex to a NC member. According to the manufacturer's instructions, the nucleic acids were detected using the chemiluminescent nucleic acid detection module (Thermo Fisher). Quantification was carried out using Image J. Error bars indicate ± SD of three replicates. *P* values were determined using a two-tailed Student's *t*-test.

#### 2.2.4 Isothermal titration calorimetry (ITC) assay

ITC was used to measure the binding affinities of YBX2-CSD with RNA oligos. All measurements were performed at 25 °C using an iTC200 (Microcal). The samples were centrifuged before each experiment to remove any protein precipitants. 0.1 μM YBX2 CSD was titrated into the experimental cell, which contained 0.01 μM RNA in 2 μL injection increments. The titration data were analyzed using the Origin 8.0 software (Microcal Software).

#### 2.2.5 Crystallization, data collection, and structure determination

The YBX2 CSD was mixed with m^5^C-RNA oligonucleotides (Takara) at a 1:2 ratio and incubated on ice for 1 h before crystallization. Crystals of YBX2 CSD (residues 85–165) and RNA oligo (UCAU(m^5^C)U) complex were grown from a solution containing 2.1 M Ammonium sulfate; crystals of YBX2 CSD (residues 85–177) and RNA oligo (UCAU(m^5^C)U) complex were grown from a solution containing 0.1 M Tris–HCl, pH 8.0, 3.2 M Ammonium sulfate; crystals of YBX2-CSD (residues 85–177) and RNA oligo-UCAU(m^5^C)UU were grown from a solution containing 30% (w/v) PEG4000, 0.1 M Tris–HCl, pH 8.5, and 0.2 M Lithium Sulfate; crystals of YBX2 CSD (residues 85–177) and RNA oligo (GU(m^5^C)U(m^5^C)) complex were grown from a solution containing 0.1 M imidazole, 20% (w/v) PEG8000, 3% (v/v) MPD. Crystals were grown at 17 °C using the hanging drop vapor diffusion method. For data collection, crystals of YBX2 CSD in complex with different RNA oligos were flash-frozen in liquid nitrogen in the above reservoir solution supplemented with glycerol. Diffraction data were collected at beamline BL18U1 of Shanghai Synchrotron Radiation Facility (SSRF). The data sets were processed using HKL3000 [25]. The structures were solved by molecular replacement with the structure of YBX1 CSD and m^5^C complex (PDB 6A6L) as a search model. Model building and structure refinement were carried out using COOT [26] and PHENIX [27]. The structural graphics were generated using PyMol (The PyMOL Molecular Graphics System, Version 2.4 Schrödinger, LLC). The statistics of data collection and refinement were summarized in Table S1. The atomic coordinates and associated structure factors have been deposited in the Protein Data Bank under accession codes 7F3I, 7F3J, 7F3K, and 7F3L.

#### 2.2.6 Droplet formation assay

Recombinant RFP-YBX2 fusion proteins were diluted from a high salt storage buffer to different concentrations in a buffer containing 20 mM Tris–HCl, pH 7.5 with indicated salt concentration. Crowding agent PEG3350 (Hampton Research) was added to a final concentration of 10% (w/v). The protein solution was immediately loaded onto a homemade chamber comprising a glass slide with a coverslip attached to two parallel strips of double-sided tape. Slides were imaged within 10 min under a Zeiss LSM 710 with a 63 × immersion objective.

#### 2.2.7 Fluorescence recovery after photobleaching (FRAP) measurements

*In vivo* FRAP experiments were carried out with a NIKONA1 microscope equipped with a 100 × oil immersion objective. Cells were cultured in a 3.5-cm laser confocal chamber and were transfected with GFP-tagged *Ybx2* plasmid 24 hrs before FRAP experiments. Droplets were bleached with a 488-nm laser pulse (2 repeats, 80% intensity, dwell time 1 s). Recovery intensity from photobleaching was recorded every 0.125 s.

*In vitro* FRAP experiments were carried out with a Zeiss LSM 710 microscope equipped with an Objectivealpha Plan-Apovhromat 63 × /1.46 Oil Corr M27 objective. Droplets were bleached with a 561-nm laser pulse (5 repeats, 100% intensity, dwell time 1 s). Recovery from photobleaching was recorded for the indicated time.

#### 2.2.8 Construction of RNA-Seq and RNA-BisSeq libraries

Bisulfite treatment for RNA-BisSeq was carried out by using EZ RNA Methylation Kit (Zymo, R5001) according to the manufacturer's instructions. RNA-Seq was carried out using HyperPre Kit (KAPA, KK8504) according to the manufacturer's instructions.

#### 2.2.9 High-throughput sequencing data pre-processing and analysis

RNA-Seq (including YBX2 RIP-Seq), RNA-BisSeq (including YBX2 RIP-BisSeq), and YBX2 PAR-CLIP-seq were carried out on the Illumina NovaSeq 6000 platform with paired-end 150 bp read length. Cutadapt (version 2.3) [28] was used to remove the adapters based on their sequence information. Trimmomatic (version 0.33) [29] was used to discard sequences with low quality and filter out reads < 35 nt in length for RNA-Seq and RNA-BisSeq and reads < 18 nt in length for PAR-CLIP-seq. (1) RNA-Seq: Clean reads were mapped to the mouse genome (mm10) using HISAT2 (version 2.0.5) [30] with default parameters. FeatureCounts (version 1.6.2) [31] was used to count the number of uniquely mapped reads (quality score ≥ 20) mapped to each gene with parameters: -t exon -g gene_id -p -s 2 -Q 20. Reads Per Kilobase per Million mapped reads (RPKM) was computed as the number of reads which map per kilobase of exon model per million mapped reads for each gene. Specifically, for YBX2 RIP-Seq, MACS2 (version 2.1.1) [32] was used for the YBX2 target peak calling with the parameters: –keep-dup all -f BAM –nomodel -g mm -B -q 0.05. The peaks were annotated by BEDTools’ intersectBed (version 2.26.0) [33]. (2) RNA-BisSeq: Clean reads were aligned to mouse genome (mm10) by meRanGh alignment (meRanTK, version 1.2.1) [34] with parameters: -fmo -mmr 0.01. meRanCall (meRanTK, version 1.2.1) [34] was used to perform m^5^C calling on the samples whose conversion ratio was over 99.5% with parameters: -mBQ 20 -mr 0 -cr 0.99 -fdr 0.05. The methylation level of each m^5^C site was calculated according to the following formula: a/(*a* + b*)*, “a” represents the number m^5^C methylation reads. “b” represents the number of unmethylated-reads (unme-reads). Only sites with coverage depth ≥ 30, methylated cytosine depth ≥ 5, and methylation level ≥ 0.1 were used for further analysis. The credible m^5^C sites were annotated by BEDTools’ intersectBed (version 2.26.0) [33]. The m^5^C distributions among 5′UTRs, CDSs, and 3′UTRs were calculated according to our previous method with an in-house Perl script. (3) PAR-CLIP-seq: Clean reads were aligned to human genome (hg19) by bowtie (version 1.2.3) [35] with parameters: –sam -v 2 -m 10 –best –strata. YBX2 target clusters were delineated by PARalyzer (version 1.1) [36] and the peaks were annotated by BEDTools’ intersectBed (version 2.26.0) [33]. We set up motif length to 5 nt, and used findMotifsGenome.pl from HOMER (version 4.11.1) [37] to analyze the YBX2 target motif with parameter: -size given -p 1 -len 5 -rna -chopify -norevopp -cache 1000.

The raw sequence data reported in this paper have been deposited in the Genome Sequence Archive [38] in the National Genomics Data Center [39], China National Center for Bioinformation / Beijing Institute of Genomics, Chinese Academy of Sciences, under accession number CRA004565 that are publicly accessible at https://ngdc.cncb.ac.cn/gsa and HRA001077 that are publicly accessible at https://ngdc.cncb.ac.cn/gsa-human.

## 3 Results

### 3.1 YBX2 is a novel RNA m^5^C binding protein

**Fig. 1:**
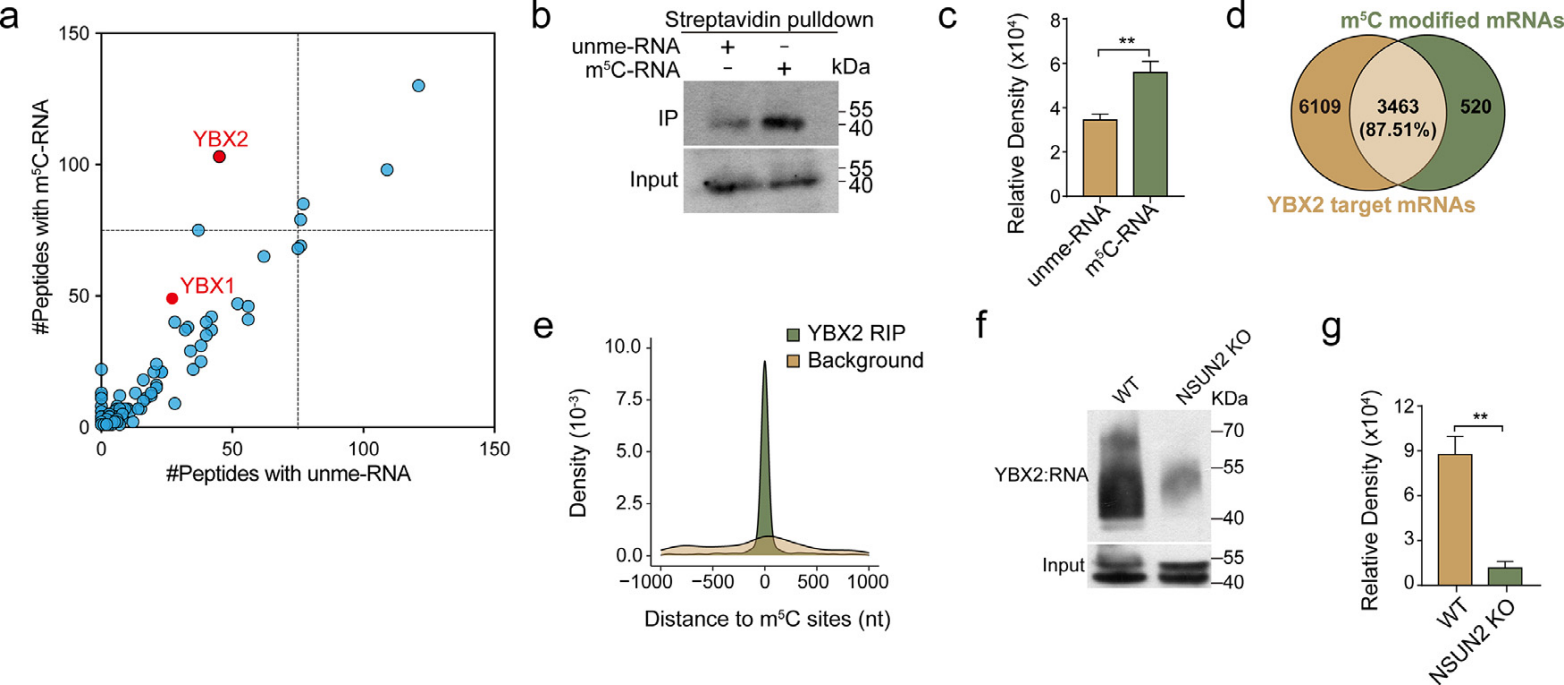
**YBX2 is a novel RNA m^5^C binding protein.** (a) Scatter plot showing the proteins bound to m^5^C-modified RNA (m^5^C-RNA) *versus* unmethylated-RNA (unme-RNA) in testis. Dashed lines mark the middle. (b) Demonstration of total testis protein lysate pulled down by biotin-labelled m^5^C-modified RNA or unmodified RNA. IP, immunoprecipitation. (c) Quantification of panel b. (d) Venn diagram showing the overlap of m^5^C-modified mRNAs and YBX2-binding mRNAs. (e) The spatial distance distribution between the YBX2-bound clusters or background clusters and m^5^C modification sites. The background is the randomly selected (*n* = 20) genome fragments that have the same length and counts with YBX2-bound clusters. (f) PAR-CLIP assay of RNA pulled down by FLAG–YBX2 in WT or *NSUN2*-deficient HeLa. YBX2-RNA indicates the band of the complex of YBX2 and RNA detected by the biotin detecting system. (g) Quantification of the gray signals of the bands in panel f. Error bars indicate ± SEM (*n* = 3).

YBX2 is specifically enriched in testis compared with other tissues in mammals [40], and shares a highly conserved CSD with the known RNA m^5^C reader YBX1 (Fig. S1a). To validate whether YBX2 could bind m^5^C-modified RNAs similarly to YBX1, we firstly carried out an oligo pull-down assay using testis total protein lysate. The unmethylated-C (unme-C) or m^5^C modified RNA bound proteins were analyzed by liquid chromatography-tandem mass spectrometry (LC-MS). The result showed that YBX2 has a high binding affinity to m^5^C-modified RNAs (Fig. 1a), which is further validated by western blot analysis of oligo pull-down products and high-performance liquid chromatography of RNA immunoprecipitation (Figs. 1b, c and S1b). We next carried out RNA-Seq and RNA-BisSeq profiling of total testis mRNAs and YBX2-bounding mRNAs, respectively. m^5^C modifications identified from our samples were enriched in the regions immediately after start codons in line with the traditional m^5^C distribution pattern (Fig. S1c). The sequencing results indicated a high overlap ratio (87.5%) between YBX2 target mRNAs and m^5^C modified mRNAs (Fig. 1d). By calculating the distance between YBX2 targets or random sequences with the same counts and length as the YBX2 target regions and m^5^C modification sites, we found that m^5^C modifications were inclined to the YBX2 binding regions (Fig. 1e). Moreover, with the analysis of YBX2 PAR-CLIP-seq in HEK293T cell line (Accession: HRA001077), we identified a “GUCUC” sequence as the YBX2 bounding motif (Fig. S1d). Integrative Genomics Viewer (IGV) tracks also demonstrated that m^5^C modifications on most genes were located within YBX2 binding regions (Fig. S1e). To further confirm that RNA binding ability of YBX2 is dependent on m^5^C modifications, we constructed a *NSUN2* KO Hela cell line (Fig. S1f, g, and h) and performed PAR-CLIP assay of YBX2. As a result, a significant decrease of RNA-binding affinity of YBX2 upon *NSUN2* depletion was detected (Fig. 1f and g). Together, these results demonstrate that YBX2 is a novel RNA m^5^C binding protein.

### 3.2 W100 is the key residue that recognizes m^5^C

**Fig. 2:**
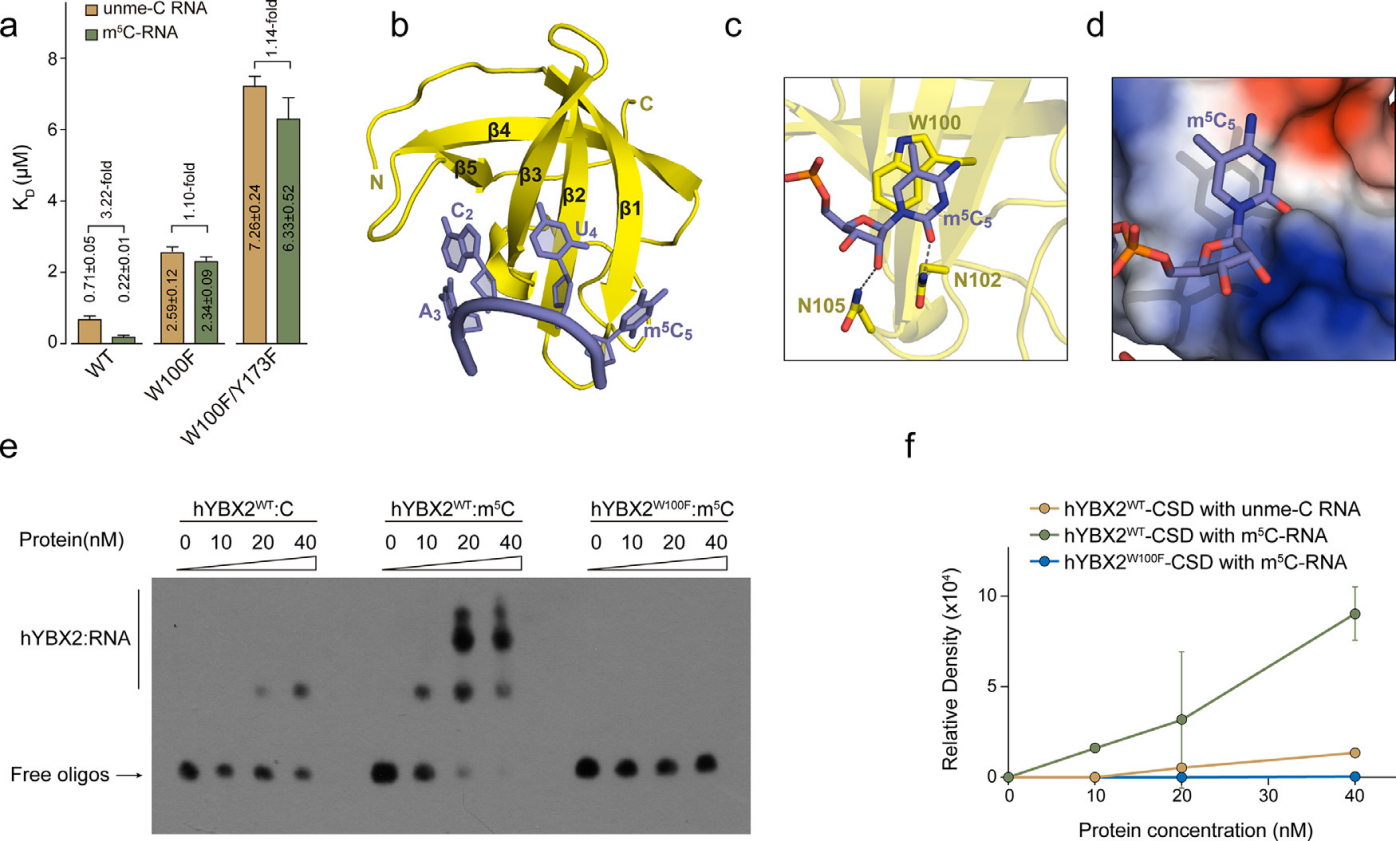
**W100 is the key residue that recognizes m^5^C.** (a) ITC measurements for the titration of wild-type or mutant hYBX2 CSD to m^5^C RNA or unme-C RNA. (b) Cartoon representation of hYBX2 CSD (yellow) in complex with RNA (slate). hYBX2 CSD represents a classical OB-fold composed of a five-stranded β-sheet. (c) Recognition of m^5^C by YBX2 CSD. Hydrogen bonds are shown in the gray dash line. (d) Electrostatic potential surface analysis of hYBX1 CSD. m^5^C RNA is bound on the surface. The color scheme from blue to red indicates the electrostatic potential from the positively charged to the negatively charged surface. (e) *In vitro* binding assay of m^5^C modified or unmodified RNA with WT or W100F mutant YBX2 CSD protein. hYBX2 indicates the human of YBX2, which is distinguished from mouse YBX2 (abbreviated to YBX2 in this article). (f) Quantification of the gray signals of the bands in the gel shift assay in (a). Error bars indicate ± SEM (*n* = 3).

YBX2 shares the same domain architecture with YBX1, including N-terminal A/P-rich domain, CSD, and CTD (Fig. S1a). We have previously reported that the CSD of hYBX1 favors m^5^C modified RNA over unme-RNA with an approx. 3.5-fold difference in affinity [13]. The sequence of the CSD of YBX2 is highly identical to that of YBX2 both in humans and mice (Fig. S2a). Therefore, we speculated that hYBX2 prefers to bind m^5^C RNA via its CSD either. We tried to use ITC to determine the binding affinity of hYBX2 to m^5^C-modified or unme-RNAs. Unexpectedly, unlike the YBX1 CSD protein, the YBX2 CSD purified protein exhibited strong phase separation properties in solution, which made it unsuitable for the RNA binding affinity measurements by ITC and crystallization trials. Therefore, based on the structure of the hYBX1 CSD and RNA complex, we generated a double mutant I92T/Q93K to successfully eliminate the phase separation properties of hYBX2 CSD, in which I92 and Q93 residues are away from the RNA binding surface to avoid interfering the binding to RNA (Fig. S2b and c). For convenience, we also referred to this mutant as wild-type in structural description and ITC binding analysis. ITC results showed that the binding affinity of hYBX2 CSD to m^5^C RNA (K_D_=0.22 ± 0.02 μM) is 3.2 times stronger than that of the unmodified RNA (K_D_=0.71 ± 0.06 μM) (Fig. 2a), consistent with the binding affinity of hYBX1 CSD to RNA where W65 in YBX1 CSD has been identified as the key residue that recognizes the m^5^C nucleotide. Next, to investigate whether W100, the corresponding residue in hYBX2 CSD, is also important for YBX2 protein binding to m^5^C, we tried to co-crystallize hYBX2 CSD and RNA complex and successfully obtained four crystal forms (Table S1). The backbones of Cα in these structures were overlapped quite well (Fig. S2d). In form I, we found that W100 π-π stacks with m^5^C RNA (Fig. 2b). N102 and N105 form hydrogen bonds with O_2_ and the 2′-OH group on the sugar ring of m^5^C, respectively, in line with the observation in the structure of YBX1 CSD-RNA (Fig. 2c). The indole ring has a strong hydrophobic interaction with the methyl group (Fig. 2d). Moreover, the EMSA experiment clearly shows that the mutation of W100 to phenylalanine (F) leads to a reduced hydrophobicity of the residue and consequently a significant decrease in binding to m^5^C (Fig. 2e and f). The ITC experiment also showed that the difference in the binding ability of the W100F mutant with m^5^C and unme-RNA was drastically reduced to about 1.1 times (Fig. 2a). In the other three complex structures (Form II-IV), we observed that m^5^C formed a π-π interaction with Y173 (Fig. S2e-g). The m^5^C nucleotide is sandwiched between N102 and Y173. Although the methyl group of m^5^C and the hydroxyl group of Y173 face the same direction, the distance between the methyl group of m^5^C and OH is about 3.5–3.8 Å beyond the weak CH—O hydrogen bond distance. To examine whether Y173 also participates in recognizing m^5^C, we generated two mutants, a single mutant W100F, and a double mutant W100F/Y173F. The binding affinity of W100F/Y173 to m^5^C (KD=6.33 ± 0.52 μM) and unme-C (K_D_=7.26 ± 0.24 μM) is 2.7 times and 2.8 times lower than that of W100F for m^5^C (K_D_=2.34 ± 0.09 μM) and unme-C (K_D_=2.59 ± 0.12 μM), respectively. The binding preference of W100F/Y173 to m^5^C or unme-C is still 1.1 times (Figs. 2a and S2h). Therefore, we conclude that W100 is the key residue that recognizes m^5^C, while Y173 is involved in RNA binding but not in m^5^C recognition.

### 3.3 YBX2 exhibits liquid droplet characteristics

**Fig. 3:**
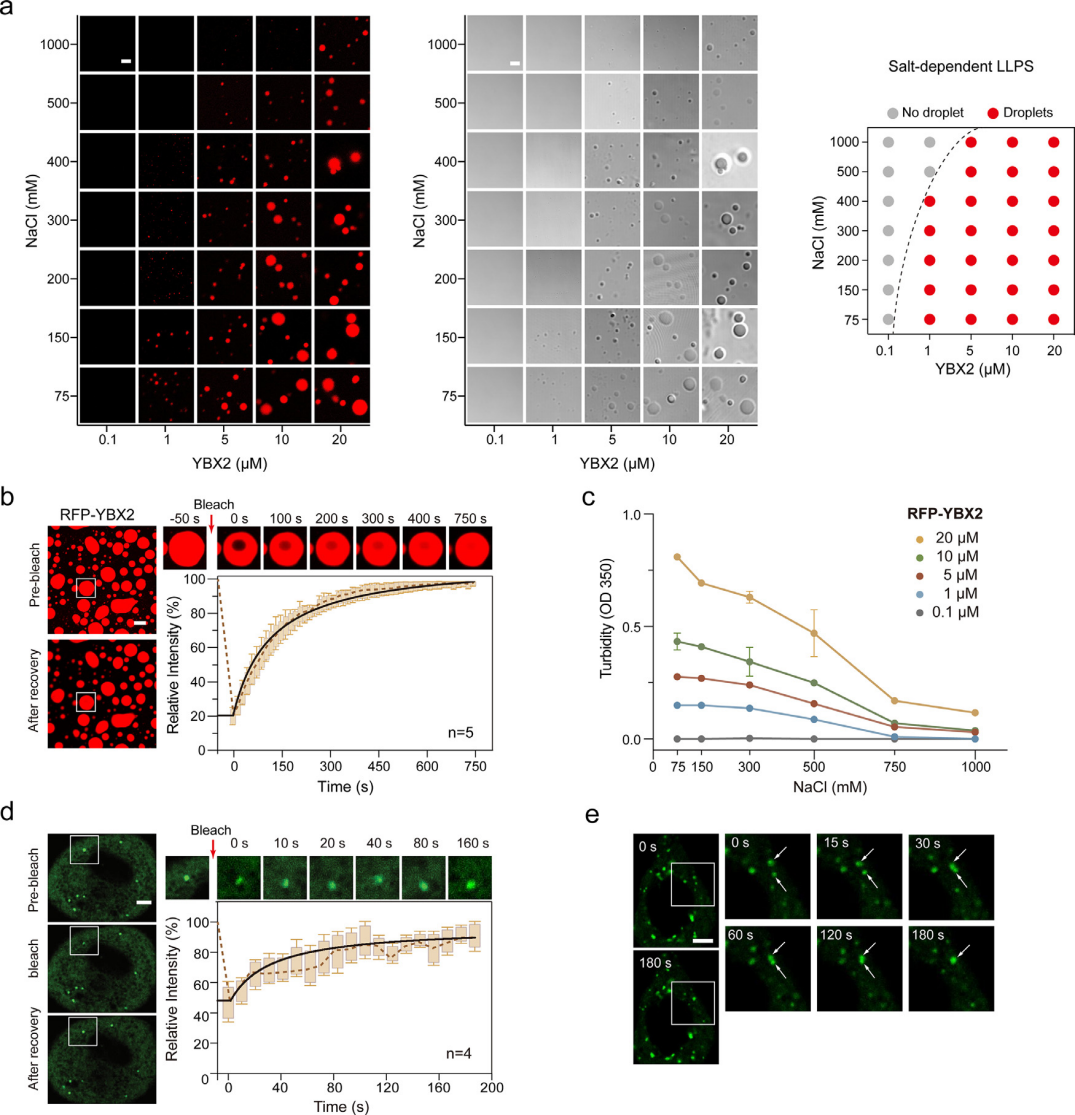
**YBX2 exhibits liquid droplet characteristics.** (a) LLPS of purified recombinant YBX2 at various amounts and with increasing concentrations of NaCl (left panel). Differential interference contrast (DIC) microscopy images show the LLPS of YBX2 (middle panel). Phase separation behaviors of YBX2 are summarized (right panel). Scale bar, 2 µm. (b) Fluorescence recovery after photobleaching (FRAP) analysis of YBX2 droplets *in vitro*. Left, representative FRAP images of YBX2 droplets; right, quantification of FRAP data. Scale bar, 10 µm. (c) Turbidity at 350 nm (OD350) was measured with increasing concentrations of NaCl. Color schemes are indicated, representing different concentrations of YBX2. (d) Top, FRAP images showing the recovery of GFP-labeled YBX2 droplets in HeLa cells. Scale bar, 5 μm. Bottom right, changes in the fluorescence intensity of GFP–YBX2 droplets after photobleaching were plotted over time. The black curve represents the mean of the fluorescence intensity in the photobleached region of interest in distinct droplets (*n* = 4); Bars indicate SEM. (e) HeLa cells overexpressed with GFP-labeled YBX2 were imaged by fluorescence microscopy for over 3 min. The fusion of GFP–YBX2 droplets can be seen in Video S3.

RBPs could play functions during the biological process through their LLPS properties. Previously reported YTHDF2 and YTHDC1, m^6^A reader proteins, could regulate stress granule formation and AML cell survival, differentiation state, and leukemogenesis through their phase separation propriety [22,41]. Because YBX2 CSD alone exhibited obvious phase separation properties, we tried to investigate whether the full-length YBX2 exhibits concentration-dependent liquid-liquid phase separation characteristics. We first fused the RFP protein to the N-terminus of the full-length YBX2 and purified the fusion protein. Fluorescence microscopy and differential interference contrast (DIC) microscopy images revealed that YBX2 could form droplets at a concentration of 1 μM (Fig. S3a and b). As the protein concentration increased or the NaCl concentration in the solution decreased, the size of these spherical droplets gradually increased (Figs. 3a, S3a, b). The fluorescence recovery after the photobleaching (FRAP) assay further showed that the fluorescence intensity of RFP-YBX2 was recovered almost 100% within 12.5 min, indicating that the YBX2 molecules in the droplet were freely exchanged with each other in the surrounding diluted solution (Fig. 3b). We also conducted a turbidity experiment of RFP-YBX2 protein and observed the formation of an opalescent solution at a concentration of 1 μM (Fig. 3c). In the crystallization trials, we found that the CSD of YBX2 exhibited phase separation properties in solution. However, the sequence prediction of the intrinsically disordered regions (IDRs) in YBX2 showed an extended IDR region at the C-terminus of YBX2 (Fig. S3c, top). Therefore, three truncated versions of YBX2 with RFP-tag fused at the N-terminus were generated, namely NTR, CSD, and CTR (Fig. S3c, bottom). Fluorescence microscopy images showed that both CSD and CTR regions could form small droplets, but not NTR (Fig. S3d). The droplets formed by the full-length YBX2 are significantly larger than those formed by CSD or CTR, indicating the contribution of both CSD and CTR to the phase separation of YBX2. A FRAP assay *in vivo* showed that the fluorescence of YBX2 droplets could recover rapidly by exchange with surrounding dilute cytoplasmic population, consistent with its *in vitro* liquid-like properties (Fig. 3d and Video S1). Through imaging studies of GFP-labeled YBX2 *in vivo*, we observed droplets fusing to form larger droplets (Fig. 3e and Video S2). Moreover, these droplets exhibited fluidity as tiny droplets merged to form larger ones over time (Fig. S3e and Video S3).

### 3.4 RNA m^5^C enhances liquid-liquid phase separation of YBX2

**Fig. 4:**
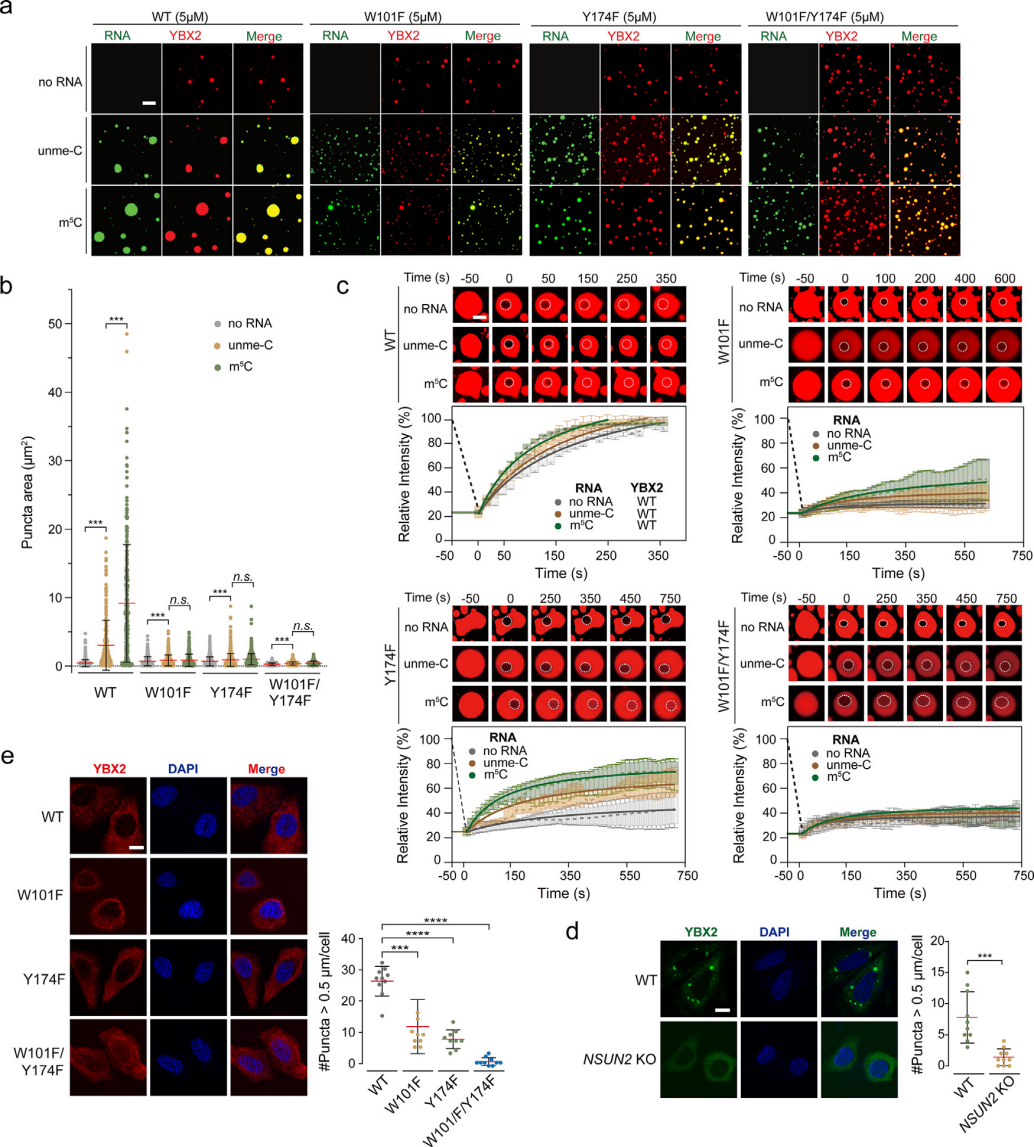
**RNA m^5^C enhances LLPS of YBX2.** (a) Representative fluorescence images of droplets formed by wild-type or mutant YBX2 proteins (red) alone or in the presence or absence of RNA (green). Scale bar, 5 μm. (b) Areas of droplets formed by wild-type or mutant YBX2 alone or in the presence or absence of RNA were counted. Error bars represent standard deviations (*n* > 280, *** *p* < 0.0001, Student's *t*-test). (c) Fluorescence recovery after photobleaching (FRAP) analysis of wild-type or mutant YBX2 droplets *in vitro*. Left panels, representative FRAP images of YBX2 droplets; right panels, quantification of FRAP data. Scale bar, 5 µm. (d) Left: Images of YBX2-tagged with GFP in WT or NSUN2 absent HeLa cells. Scale bar = 5 μm. Right: The number of YBX2-GFP puncta > 0.5 µm in each cell was assessed by Image J. Statistical analysis was performed with unpaired/two-tailed T-tests. Data are shown as mean ± SEM. ***p < 0.0001. (E) Left: Images of WT or m^5^C binding sites mutant YBX2 in WT HeLa cells. Scale bar = 5 μm. Right: The number of YBX2-GFP puncta > 0.5 µm in each cell was assessed by Image J. Statistical analysis was performed with unpaired/two-tailed T-tests. Data are shown as mean ± SEM. ***p < 0.0001, ****p < 0.00001.

It is worth noting that m^5^C modification enhances the binding of YBX2 to RNA, which prompted us to test whether m^5^C could promote the phase separation of YBX2 *in vitro*. YBX2 showed obvious LLPS at very low concentrations (∼5 μM) *in vitro*. Then we evaluated the droplet formation of WT or mutant YBX2 under this concentration by mixing iFluor488-labelled RNA with or without m^5^C modification with iFluor561-labelled YBX2 at a 1:1 ratio. Under the same protein concentration, the number and size of droplets formed by the YBX2-m^5^C complex were much larger than those formed by YBX2-unme-C or YBX2 alone (Fig. 4a and b). In contrast to the observations for wild-type YBX2 and RNA, the droplet size formed by W101F mutant with either unme-C or m^5^C RNA complexes did not seem to change significantly. However, the statistics of the droplet sizes showed that the droplet formed by the W101F-m^5^C complex was still larger than its unme-C complex and even larger than W101F alone. Y174F and the double mutant W101F/Y174F shared a similar behavior as W101F ( Fig. 4a and b).

Consistent with biochemical data, W101F and W101F/Y174F mutants, which disrupt the binding preference of m^5^C over unme-C, effectively attenuated the LLPS of the YBX2-m^5^C complex, demonstrating the important role of m^5^C recognition in the formation of YBX2 droplets (Fig. 4c). *In vivo* GFP-tagged YBX2 phase separation assay in wild-type and NSUN2 absent cells illustrated a defective phase separation ability of YBX2 in the absence of RNA m^5^C, supporting the critical role of m^5^C modification for YBX2 phase separation (Fig. 4d). *In vivo* YBX2 droplet formation assay showed that YBX2 phase separation is only attenuated by the loss of one m^5^C binding site, but totally disturbed after both m^5^C binding sites were mutated. These data confirmed that m^5^C binding ability is critical for YBX2 phase separation (Fig. 4e).

## 4 Discussion

In this study, we identified YBX2 as a new RNA m^5^C binding protein and W100 (W101 in mouse) is the key residue responsible for its recognition of m^5^C, illustrating a similar functional mode as YBX1. Moreover, in addition to the four known conserved aromatic residues that mainly contribute to the binding of YBX2 to RNA, we also identified another conserved aromatic residue, Y173 (Y174 in mouse), plays roles in the RNA binding. Although Y173 is located on a loop, judging from the superposition of the four structures, the loop is rigid. A groove is also formed between Y173 and N102, suitable for the insertion of a nucleotide to form a sandwich structure. In crystal form II-IV, we observed that m^5^C contacts Y173 through π-π interactions. However, after being validated by ITC binding experiments, it is likely to be caused by crystal packing. By mutating Y173 to F, only a marginal difference of the binding affinity between m^5^C or unme-RNA was exhibited compared to W100F mutant. These data further point to a critical role of W100 in m^5^C recognition, and meanwhile, suggest a potential mechanism of CSD-mediated RNA binding beyond YBX family proteins.

Phase separation of biomacromolecules such as proteins, RNAs, and DNAs has been reported to be involved in many biological processes, including cell transcription [42], translation [43], and RNA splicing [44]. The formation of coagulants or membraneless organelles can make these macromolecules spatially ordered with dynamic switch among different states [45]. In our study, YBX2 tends to form phase separation both *in vitro* and *in vivo*. Upon binding to RNA, the puncta size increased dramatically. Most importantly, we observed that m^5^C could further enhance the phase separation of YBX2. YBX2 is specifically expressed in testis from the pachytene stage and peaks at the round spermatid stage, accounting for about 0.7% of total protein in male germ cells [40], suggesting a dynamic regulatory role of YBX2 in spermatogenesis. Chromatoid bodies are identified as an RNA storage and processing center in the cytoplasm of haploid spermatids. Many RNA binding proteins such as those involved in the piRNA pathway or nonsense-mediated decay (NMD) pathway are components of chromatoid bodies [46]. Interestingly, NSUN2 is also located in the chromatoid body [2]. Upon KO of the m^5^C writer *NSUN2*, the puncta formed by GFP-YBX2 diminished, indicating that m^5^C modification plays an important role in YBX2 phase separation.

Considering that YBX2 shows a specific and dynamic expression pattern in mouse testis, we propose that YBX2 could regulate spermatogenesis through m^5^C regulated phase separation. Further elucidation of the role of phase separation in cell and RNA fate determination will help us understand the underlying molecular mechanism for the pathogenesis of various diseases and provide a theoretical basis for designing new diagnostic and therapeutic modalities. Moreover, in corroboration with previous findings [2], the potential clinical use of phase separation in male infertility is worthy of further investigation.

## 5 Conclusion

Overall, our work illustrated YBX2, a homologous protein of YBX1, acts as a novel RNA m^5^C binding protein, and could bind to m^5^C-modified RNAs through its W100 position (W101 for mouse), with Y173 position (Y174 for mouse) as the auxiliary binding site. More intriguingly, YBX2 mediates liquid-liquid phase separation that is augmented by RNA m^5^C. Thus, our findings provide important mechanistic insights into the interaction between RNA m^5^C methylation and phase separation, enriching our current understanding of epigenetic regulation during biological processes.

## Declaration of Competing Interest

The authors declare that they have no conflicts of interest in this work.
