# Supplementary material for: RNA 5-methylcytosine regulates YBX2-dependent liquid-liquid phase separation
Source: Fundam Res. 2021 Dec 8;2(1):48–55. doi: 10.1016/j.fmre.2021.10.008 (PMC11197489; doi:10.1016/j.fmre.2021.10.008)
Supplement: Supplementary file 1 [file mmc1.docx]

**Supplementary Figures and Figure legends**


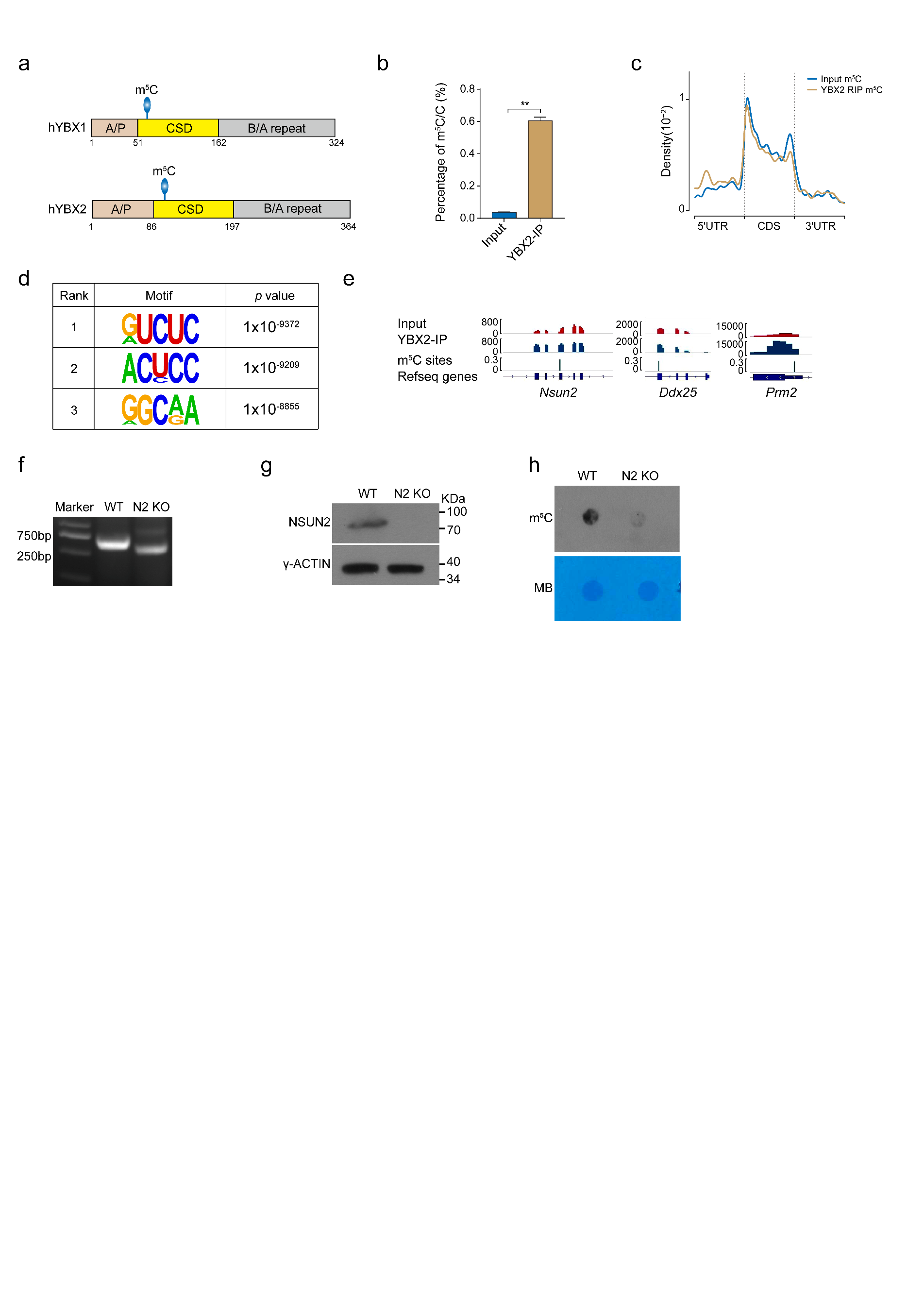


**Fig. S1 | YBX2 is a novel RNA m^5^C binding protein.** (a) Schematic diagram showing the domain architectures of YBX1 and YBX2 protein. (b) The bar graph shows the percentage of m^5^C-modified RNA in YBX2 bound RNAs or input from UHPLC-MS/MS. Error bars indicate ±SEM (n = 3). (c) Density plot showing the distribution pattern of m^5^C sites along with mRNA transcripts of input and YBX2-bounding RNAs. (d) Sequence motifs of YBX2 bounding regions on mRNAs. (e) Integrative Genomics Viewer (IGV) plot showing the input and YBX2 target peaks and m^5^C sites on *Nsun2*, *Ddx25* and *Prm2*. (f) Detection of the NSUN2 knock-out result using PCR. (g) Western blot results showing the successful depletion of NSUN2 in HeLa. (h) Dot blot result showing the m^5^C level decrease significantly in NSUN2 knock-out cells.


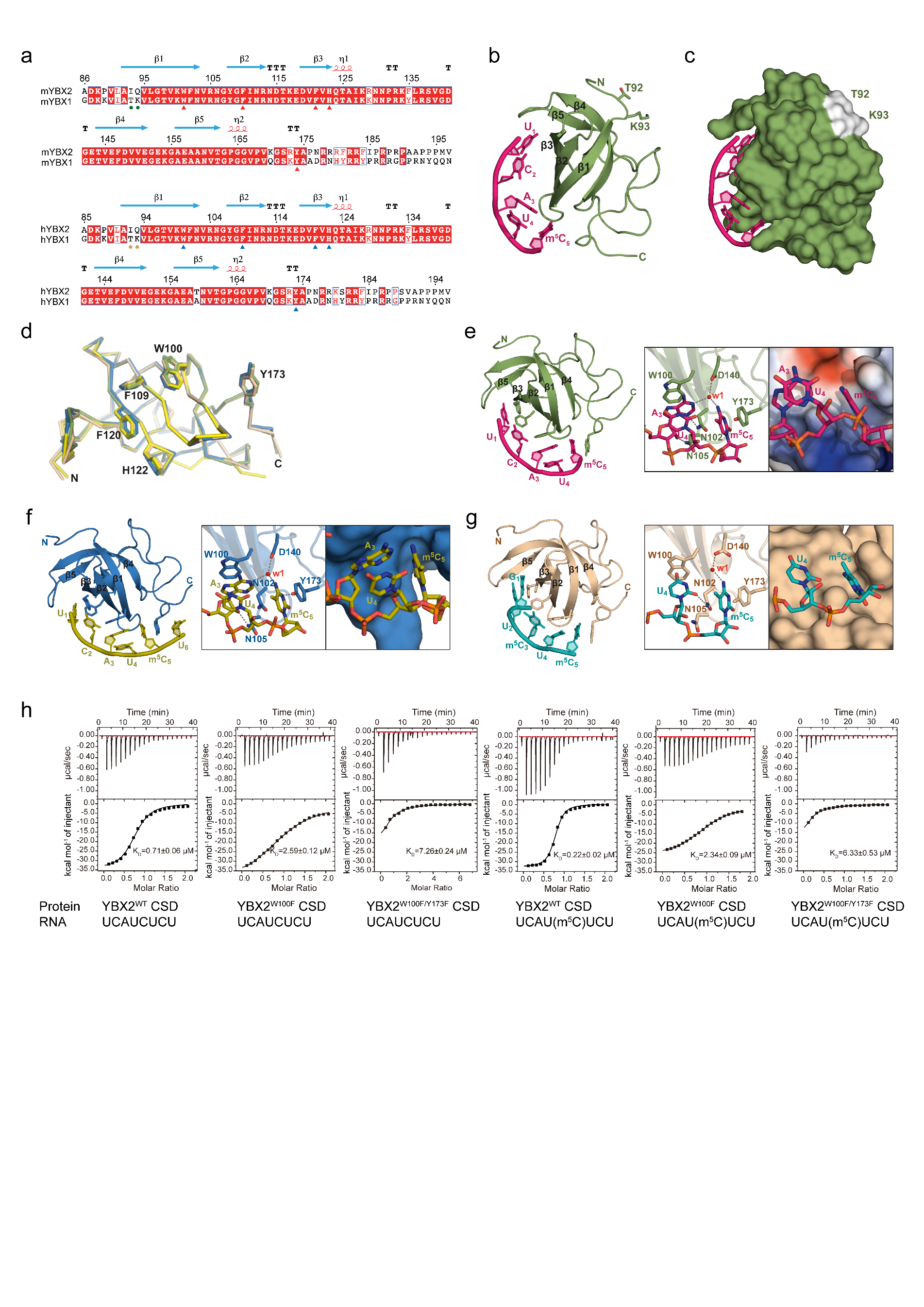


**Fig. S2 | W100 is the key residue that recognizes m^5^C.** (a) Sequence alignment of YBX2 CSD homologs, including human (hs, *homo sapiens*, P67809), mouse (mm, *mus musculus*, P62960), Identical residues are shaded in red. Secondary structural elements of YBX2 CSD are displayed above. Red triangles mark the residues involving π-π stacking interactions. Point mutation sites I93 and Q94 are labelled in green circles. (b)-(c) Overall structure and Electrostatic potential surface analysis of hYBX2 CSD (green) in complex with RNA (hot pink), respectively. T92 and K93 are away from the RNA binding site. (d) Superimposition of the Cɑ backbones hYBX2 CSD in four crystal forms. (e)-(g) Structures of hYBX2 CSD in complex with RNA in crystal form II-IV. Overall structures are shown in the left panels. Interaction details are shown in the middle panels. The right panels show that RNAs (stick mode) binding to the surface of hYBX2. Hydrogen bonds are shown in gray dash line. (h) Raw data of ITC measurements are shown as injection profiles (top panels) and the calorimetry binding isotherm (bottom panels). The association constant (K_D_) for each binding curve was indicated.


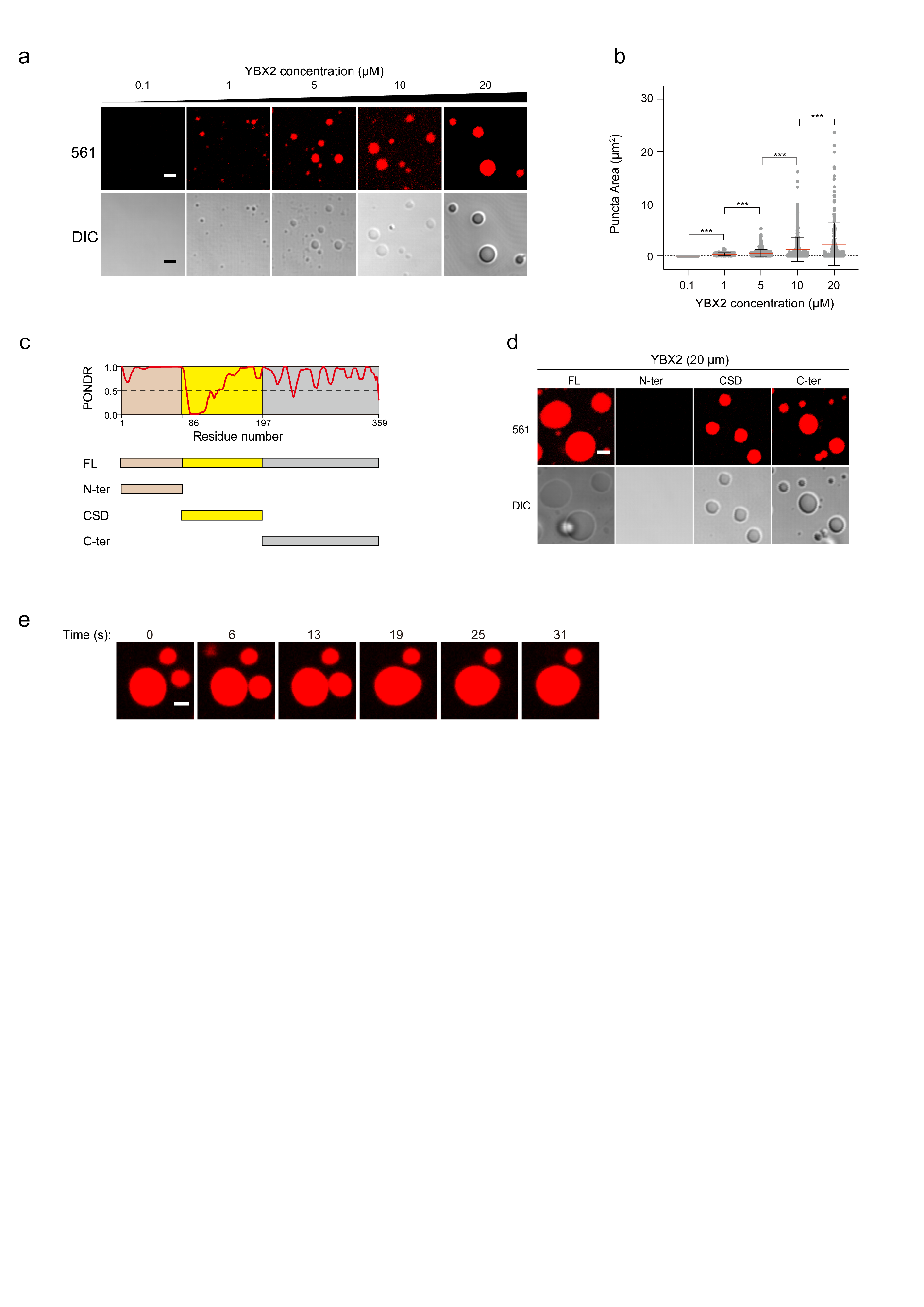


**Fig. S3 | YBX2 exhibits liquid droplet characteristics.** (a) Fluorescence (top) and DIC (bottom) images of YBX2 at indicated concentrations under 150 mM NaCl, 20 mM Tris-HCl, pH 7.4, 10% (w/v) PEG 3350. (b) Count statistics of droplets area formed by YBX2. Error bars represent standard deviations (n>200, *** *p*<0.0001, Student’s t-test). (c) Prediction of IDR regions in YBX2 by PONDR (top). Schematic view of full-length and truncated versions of YBX2 (bottom). (d) Behaviors of phase separation of full-length and truncated versions of YBX2. (e) Fluorescence images of the fusion of YBX2 droplets.
